# Geographic shifts in climatically suitable areas and loss of genetic variability under climate change in a neotropical tree

**DOI:** 10.1186/1753-6561-5-S7-O4

**Published:** 2011-09-13

**Authors:** Jose Alexandre Diniz-Filho, Rosane Collevatti, Lázaro Chaves, Thannya Soares, João Carlos Nabout, Thiago Fernando Rangel, Dayane Melo, Jacqueline Lima, Mariana Telles

**Affiliations:** 1 Departamento de Ecologia, ICB, Universidade Federal de Goiás, Brazil; 2 Departamento de BIologia Geral, ICB, UFG, Brazil; 3 Escola de Agronomia & Engenharia de Alimentos, UFG, Brazil; 4 UnUCET, Universidade Estadual de Goiás, Brazil; 5 Departamento de Ecologia, ICB, UFG, Brazil; 6 Programa de Pós-Graduação em Agronomia, UFG, Brazil; 7 Programa de Pós-Graduação em Ecologia & Evolução, UFG, Brazil

## Background

Many species are expected to suffer a strong shift in geographic ranges due to climate changes in the next fifty years, depending on their ecological tolerance and current demographical parameters, which were in turn shaped by their evolutionary history. These shifts may also cause a change in genetic population structure and variability, because local extinctions or reduction in fitness are not expected to be random in geographical space. Here we used an ensemble forecast approach of Species Distribution Modeling (SDM hereafter, also known as niche modeling) to derive current and future geographic distribution of the Neotropical tree *Dipteryx alata* (“Baru” tree, Fabaceae). We then obtained a series of genetic parameters for the species after generating extinctions in areas of low future habitat suitability.

## Methods

We obtained a total of 448 occurrences of *D. alata* throughout the Brazilian Cerrado, which were recorded in a grid with 6240 cells of 0.5^o^ of latitude/longitude covering South America. These occurrences were modeled as a function of eight climatic variables (WORLDCLIM), for the current time and projected into2050,for three different Global Circulation Models (AOGCMs - CCCma, Csiro, HadCm3) [1,2].

Occurrences were modeled using six different SDM techniques [3]. Methods used were BIOCLIM, Euclidian, Gower and Mahalanobis distances, GARP and MAXENT. For each of these methods, models were built using 255 combinations of the climatic variables, each one tested using 50 cross-validations using True-Skill statistics (models with TSS < 0.7 were excluded). A Principal Component Analysis (PCA) of the estimated frequencies of occurrence, obtained by SDMs and AOGCMs, was used to compare the maps. Variance components of these sources were obtained and mapped [2].

Genetic data for *D. alata* consisted in microsatellites markers analyzed for 25 widely distributed local populations, encompassing species’ geographical range. A total of 644 individual trees were genotyped for eight microsatellite loci. Genetic parameters were used to estimate the total amount of polymorphism and genetic diversity currently found in *D. alata*. We estimated the number of alleles per locus, genetic diversity (expected heterozygosity under Hardy–Weinberg equilibrium), and the expected heterozygosity under mutation-drift equilibrium. Moreover, we recalculated these parameters by assuming that, under climate change, there will be a displacement of climatically suitable areas for the species and, consequently, that only populations found in regions above certain levels of suitability in the future will persist.

## Results and discussion

**Figure 1:**
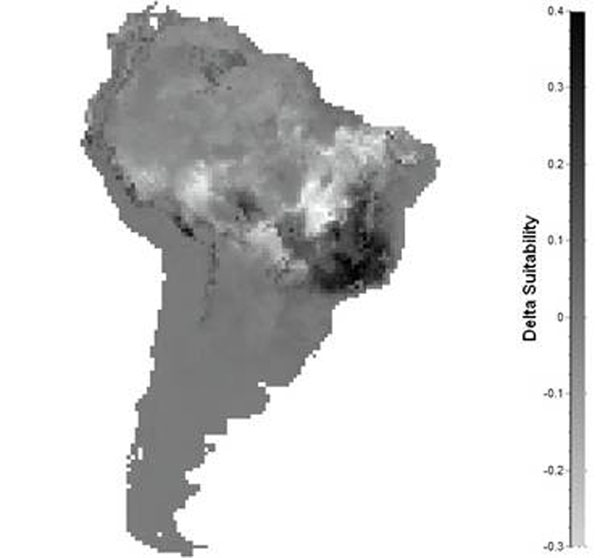
Shifts of climatically suitable areas in 2050 for *Dipteryx alata*, expressing change in estimated frequency of occurrences of the species based on six species distribution models projected into three GCMs.

The ensemble forecasting approach reveals that *D. alata* will shift its geographic range and climatically suitable areas from Central towards Southeastern Brazil (Fig. 1). The first principal component explains 74.3% of the variation among maps and, on average, 95% of the variation among them is due to SDMs used.

**Figure 2:**
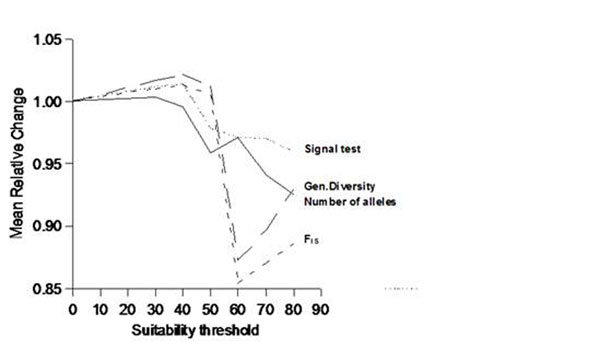
Reduction in genetic parameters of *D. alata* estimated at increasingly levels of environmental suitability estimated for 2050.

The changes in the climatically suitable areas in *D. alata* imply a reduction in the genetic parameters. The parameters remain approximately constant up to a 50% threshold, which is the minimum by assuming a majority consensus of frequency of occurrences. However, after this critical threshold there is an abrupt reduction in all parameters, although the magnitude of shift is only about 10% of the current values, on average (Fig. 2). There is a wide variation of shifts direction and magnitude among loci for each parameter, and actually these trends are usually driven by two or three loci.

The wide current range and ecological tolerance of *D. alata* explains why low levels of loss in genetic diversity were observed here, contrasting with previous results for other species (i.e., the “pequi” tree *Caryocar Braziliense*). Because of the wide range, currently and in the future, some of the local populations with highest genetic diversity will potentially remain in highly suitable areas in the future, even using very conservative thresholds of 80%. However, it is important to highlight that coalescence analyses and the expected heterozygosity under mutation-drift equilibrium suggest strong population bottlenecks for the species in the recent past, which were corroborated by hind cast projections of the SDMs using paleoclimatic data. This explains the low level of genetic variability *in D. alata*, despite its wide geographic range.

Thus, despite the shift in geographic range size and climatically suitable areas towards Southeastern Brazil and the expected downward shift in the genetic parameters, the analyses performed here do not show a strong loss of genetic diversity in *D. alata*. Even so, it is important to realize that these results are mainly due to the relatively low current genetic variability of the species, probably associated with recent population bottlenecks. In this case, climatic shifts can have more serious adaptive consequences and further investigations are necessary to avoid a false indicative of high probability of persistence of this species based on a low relative level of loss of genetic diversity.
